# Nursing allocation in isolation wards of COVID-19 designated hospitals: a nationwide study in China

**DOI:** 10.1186/s12912-021-00795-w

**Published:** 2022-01-19

**Authors:** Hong-fei Ren, Feng-jiao Chen, Ling-xiao He, Chang-qing Liu, Ying-ying Liu, Yu-jia Huang, Hui Han, Su Fu, Ming-guang Zhang, Yan Jiang

**Affiliations:** 1 West China School of Nursing, Sichuan University, NO. 37 Guoxue Alley, Wuhou District, Chengdu, 610041 Sichuan Province China; 2 Department of Gastroenterology, West China Hospital, Sichuan University, NO. 37 Guoxue Alley, Wuhou District, Chengdu, 610041 Sichuan Province China; 3 Hematology Department of West China Hospital, Sichuan University, NO. 37 Guoxue Alley, Wuhou District, Chengdu, 610041 Sichuan Province China; 4 Trauma Center of West China Hospital, Sichuan University, NO. 37 Guoxue Alley, Wuhou District, Chengdu, 610041 Sichuan Province China; 5 Operating Room of Anesthesia Surgery Center, West China Hospital, Sichuan University, No. 37, Guoxue Alley, Wuhou District, Chengdu, 610041 Sichuan China; 6 Nursing Key Laboratory of Sichuan Province, Sichuan University, No. 37, Guoxue Alley, Wuhou District, Chengdu, 610041 Sichuan China; 7 Neuro General Ward of West China Hospital, Sichuan University, NO. 37 Guoxue Alley, Wuhou District, Chengdu, 610041 Sichuan Province China; 8 Nursing Department of West China Hospital, Sichuan University, NO. 37 Guoxue Alley, Wuhou District, Chengdu, 610041 Sichuan Province China

**Keywords:** Bed-to-nurse ratio, COVID-19, Designated hospital, Nursing workforce allocation, Nurse work hours per shift

## Abstract

**Background:**

Appropriate allocation of nursing staff is key to ensuring efficient nursing in hospitals, and is significantly correlated with patient safety, nursing quality, and nurse job satisfaction. However, there are few studies on nursing workforce allocation in the isolation wards of COVID-19 designated hospitals globally. This study aims to better understand the nursing workforce allocation in the isolation wards of COVID-19 designated hospitals in China, and provide a theoretical basis for efficiently deploying first-line nurses in China and across the world in the future.

**Methods:**

An online survey was conducted among the head nurses (*n* = 229) and nurses (*n* = 1378) in the isolation wards of 117 hospitals (selected by stratified sampling), using a self-reported human resource allocation questionnaire.

**Results:**

The average bed-to-nurse ratios of different isolation wards were different (Z = 36.742, *P* = 0.000). The bed-to-nurse ratios of the ICU, suspected COVID-19 cases ward, and confirmed COVID-19 cases ward, were 1:1.88, 1:0.56, and 1:0.45, respectively. The nurse work hours per shift in different isolation wards were also different (Z = 8.468, *P* = 0.014), with the specific values of the ICU, suspected COVID-19 cases ward, and confirmed COVID-19 cases ward, being 5, 6, and 6 h, respectively. A correlation analysis showed that the average work hours per shift was proportional to the overtime work of nurses (r_s_ = 0.146), the proportion of nurse practitioners was proportional to the overall utilization rate of nursing human resources in the wards (r_s_ = 0.136), and the proportion of nurses with college degrees was proportional to teamwork (r_s_ = 0.142). The proportion of nurses above grade 10 was inversely proportional to teamwork and psychological problems (r_s_ = 0.135, r_s_ = 0.203). The results of multiple stepwise regression analyses showed that the work hours of nurses per shift was the main factor affecting nurse satisfaction and that the proportion of nurses and the work hours of nurses per shift were both independent factors affecting the length of stay (LOS) of patients.

**Conclusion:**

Hospitals in China have made good nursing workforce allocations during the COVID-19 pandemic, but there are certain shortcomings. Therefore, scientific and efficient nursing workforce allocation practice plans should be established to improve the ability of hospitals to deal with public health emergencies and are urgent problems that need to be addressed soon.

## What is already known

- Nursing workforce allocation is significantly correlated with patient safety, nursing quality, and nurse job satisfaction.
- There are no criteria on nursing workforce allocation in the isolation wards of COVID-19 designated hospitals globally.

## What this paper adds

- The bed-to-nurse ratios of isolation wards differ. In detail, they are 1:0.56 in suspected cases wards, 1:0.45 in confirmed cases wards, and 1:1.88 in confirmed severe cases wards (ICUs).
- The average nurse work hours per shift of the ICU, suspected COVID-19 cases ward, and confirmed COVID-19 cases ward are 5, 6, and 6 h, respectively.
- The work hours of nurses per shift is the main factor affecting nurse satisfaction. Furthermore, the proportion of nurses and the work hours of nurses per shift are both independent factors affecting the length of stay (LOS) of patients.

## Background

COVID-19 (Corona Virus Disease 2019) is an acute respiratory infectious disease that is mainly transmitted by contact with respiratory droplets [1]. Since December 2019, the pandemic has spread globally and has the characteristics of being rapid, causing great harm, and being extensive, which seriously threatens public safety and society. On January 20, 2020, China’s Health Commission stipulated that COVID-19 control would require measures for the prevention and control of “Class A” infectious diseases [2]. On January 30, 2020, the World Health Organization (WHO) listed the COVID-19 pandemic as a major international public health emergency [3]. To date, there has been a total of more than 169 million confirmed cases and 3.5 million deaths from this pandemic. It has become the largest public health emergency in the world in more than a century.

In the face of COVID-19 outbreaks, hospitals, as the main battlefields, undertake the important tasks of screening suspected cases, diagnosing and treating confirmed cases, and rescuing critical cases. As a result, nurses on the frontlines are facing greater challenges. On the one hand, the demand for nursing staff and their proficiency are rising globally, while on the other hand, the accuracy and timeliness of nursing workforce allocation have increased requirements. In addition, considering the specifics of COVID-19 disease and the great need for prevention and control, the nursing workload has greatly increased and nursing workforce allocation has been severely challenged.

To our knowledge, having enough nurses with manageable workloads is beneficial to patients, medical staff, and hospital operations. It has been verified that there is a significant correlation between the scientific allocation of nursing human resources and the sensitive indexes of nursing quality, patient safety, patient mortality, and job satisfaction [4, 5]. Among these, it is noteworthy that nurse job satisfaction should be considered as one key evaluation metric of nursing allocation since it was verified that nursing staff level (e.g., nurse to patient ratio, nurse working hours, and nurses skill mix) was associated with nurse job satisfaction [6]. Besides, evidence continues to show that better hospital nurse staffing is associated with a shorter length of stay (LOS). In detail, nurse staffing improvements by allocating one patient per nurse resulted in reductions in LOS (IRR = 0·97, *p* = 0·035) [7]. Hence, to ensure efficient nurse staffing, it is necessary to understand the staffing level of frontline nursing staff in COVID-19 designated hospitals and its relationship with nurse satisfaction and LOS.

At present, there are few studies on the allocation of the nursing workforce in the isolation wards of COVID-19 designated hospitals globally. Previous research has only suggested that COVID-19 patients undergoing mechanical ventilation should have assigned nursing based strictly on a nurse to patient ratio of 1:1 [8]. One study [9] has preliminarily discussed nursing workforce allocation methods that can be adopted for the COVID-19 pandemic, and another study [10] has investigated the actual work hours and expected work hours of frontline nurses in each shift. However, there is a lack of large sample surveys on overall nursing workforce allocation in the isolation wards of COVID-19 designated hospitals in China.

Therefore, the purpose of this study is to assess: 1) the present situation and evaluate nursing workforce allocation in isolation wards (i.e., suspected case isolation, confirmed case isolation, and ICU wards), 2) the job satisfaction of front-line nursing staff, and 3) the relationship between nursing staffing and LOS, during the COVID-19 pandemic. This would provide a reference for nursing workforce allocation in COVID-19 designated hospitals in China. It also provides an important reference for scientifically and efficiently deploying the frontline nursing workforce in China and globally, for future public health emergencies.

## Methods

### Participants

In this study, 132 COVID-19 designated hospitals from 31 provincial administrative districts in China were selected using stratified sampling. The head nurses and nurses in the isolated wards of these hospitals were asked to complete a self-reported nursing human resource allocation questionnaire that includes three parts: (1) informed consent form; (2) general data of hospitals and participants; (3) evaluation of the status quo and its effect on the isolated nursing workforce allocation. The inclusion criteria for the participants were as follows: (1) being the head nurse or a nurse in the isolation ward of a COVID-19 designated hospital, and being engaged in caring for COVID-19 patients for more than 1 month; (2) having volunteered to participate in this study.

### Procedure

From April 11 to July 31, 2020, a retrospective cross-sectional online survey to explore frontline nursing staff allocation in the isolation wards of COVID-19 designated hospitals was conducted. First, participants provided informed consent on the survey platform before they could proceed to the survey items. Next, the questionnaire was sent to each participant via a mobile phone text message or Wechat, and the respondent could only complete the questionnaire once to avoid repeated submissions. One week after the questionnaire was sent, the researcher verified the number and quality of completed questionnaires. In case of any ambiguities, the researcher could contact the participants by telephone to verify the accuracy of the data.

### Statistical analysis

Quantitative data were presented as means or medians with interquartile ranges as appropriate, whereas categorical data were presented as frequencies and corresponding ratios. Differences in nursing staff level among different wards were analyzed using the Kruskal-Wallis Test, while relationships between the nursing staff level and effect evaluation index (e.g., rate of fatigue, medical staff satisfaction) were assessed using Spearman or Pearson correlation analyses as appropriate. Independent predictors of nurses’ satisfaction or patients’ length of hospitalization were then identified via logistic regression analyses. All statistical analyses were conducted using SPSS 16.0 (IBM, NY, USA), and statistical significance was based on a *p*-value of 0.05.

## Results

### Characteristics of participants

In this study, a total of 229 head nurses and 1378 nurses from 117 COVID-19 designated hospitals were surveyed. Among head nurses, 223 (97.4%) were women with a median age of 39 years, 85.2% had a bachelor’s degree, 55.5% had a professional title of ‘nurse supervisor’, and the median length of employment was 20 years. Among frontline nurses, 1285 (93.3%) were women with an average age of 31.48 years, 76.0% had a bachelor’s degree, and 76.0% had a professional title of ‘nurse’. More details are presented in Table 1.

**Table 1:** General information of participants in isolation wards of COVID-19 designated hospitals

Items	Classification	N,%	
		Head nurse (*n* = 229)	First-line nurse (*n* = 1378)
Age (years)	<30	2 (0.9)	680 (49.3)
	30–39	114 (49.8)	612 (44.4)
	40–49	106 (46.3)	71 (5.2)
	≥50	7 (3.1)	15 (1.1)
Sex	Male	6 (2.6)	93 (6.7)
	Female	223 (97.4)	1285 (93.3)
Ethnicity	Han	202 (88.2)	1240 (90.0)
	Minority	27 (11.8)	138 (10.0)
Educational level	Junior college	25 (10.9)	1047 (76.0)
	Undergraduate	195 (85.2)	314 (22.8)
	Graduate degree	9 (3.9)	17 (1.2)
Professional title	Nurse	19 (8.3)	1047 (76.0)
	Supervisor nurse	127 (55.5)	314 (22.8)
	Associate professor or above	83 (36.2)	17 (1.2)
Hospital level	Level III hospital	195 (85.1)	1219 (88.4)
	Level II hospital	34 (14.9)	159 (11.6)
Hospital category	General hospital	144 (62.9)	1109 (80.5)
	Specialized Hospital	85 (37.1)	269 (19.5)
COVID-19 designated hospital level^a^	Provincial level	104 (45.4)	517 (37.5)
	Municipal level	78 (34.1)	546 (39.6)
	District level	47 (20.5)	315 (22.9)
Isolated ward category	Suspected case ward	65 (28.4)	370 (26.9)
	Confirmed mild case ward	8 (3.5)	116 (8.4)
	Confirmed mixed case ward	119 (52.0)	637 (46.2)
	Confirmed severe case ward (ICU)	37 (16.2)	225 (16.3)

^a^A hospital that has been designated by the National Health Commission of the People’s Republic of China to receive and cure COVID-19 patients

### Nursing workforce allocation in different types of isolation wards

From Table 2, it can be seen that: (1) the average bed-to-nurse ratio of different types of isolation wards was different (Z = 36.742, *P* = 0.000). Multiple comparisons show that there was no statistically significant difference in bed-to-nurse ratio between the wards of suspected cases and confirmed cases (1:0.56 vs 1:0.45, *P* = 0.077). However, differences in other pairwise comparisons were statistically significant, among which the bed-to-nurse ratio in the ICU ward was the highest, with an average of 1:1.88; (2) work hours of nurses per shift in different types of isolation wards were different (Z = 8.468, *P* = 0.014). Multiple comparisons showed that there was no significant difference in the average work hours per shift between the suspected case wards and confirmed case wards (*P* = 0.433), but differences in other pairwise comparisons were all statistically significant. The average work hours per shift in the severe ICU ward was 5 h, while in the suspected case and confirmed case wards were both 6 h; (3) in terms of nursing staff structure, there was a significant difference in the ratio of nurses with junior college degrees in different wards (Z = 8.693, *P* = 0.013). Multiple comparisons showed that there was no significant difference in the proportion of nurses with junior college degrees between the suspected case and confirmed case wards (40% vs 38%, *P* = 0.256), but differences in other pairwise comparisons were all statistically significant. The ratio of nurses with junior college degrees in the severe ICU ward was 20%, and the proportion of nurses with bachelor’s degrees was as high as 73%.

**Table 2:** Nursing workforce allocation in different types of isolation wards

	M (P75 - P25)			Statistical value	P
Items	Suspected case ward	Confirmed case ward	Confirmed severe case ward (ICU)		
Average bed-to-nurse ratio	1:0.56 (0.58)	1:0.45 (0.47)	1:1.88 (1.83)	36.742	**0.000**
Average shift hours (h)	6 (2)	6 (4)	5 (4)	8.468	**0.014**
Average weekly rest time per person (d)	2 (0)	2 (1)	2 (1)	4.006	0.135
Primary nurse ratio (%)	27 (31)	27 (25)	21 (24.5)	2.863	0.239
Senior nurse ratio (%)	43 (29.5)	43 (26)	50 (16.5)	4.188	0.123
Supervisor nurse ratio (%)	17 (19.5)	20 (16)	18 (19.5)	3.066	0.216
Associate professor or above ratio (%)	0 (7.5)	3 (8)	2 (7)	0.157	0.925
Junior college degree ratio (%)	40 (43.5)	38 (47)	20 (28.5)	8.693	**0.013**
Undergraduate degree ratio (%)	58 (44.5)	62 (43)	73 (38)	5.683	0.058
Graduate degree ratio (%)	0 (0)	0 (0)	0 (1)	2.771	0.250
Proportion of 0–2 working years (%)	9 (18.5)	6 (14)	8 (15.5)	3.548	0.170
Proportion of 3–5 working years (%)	25 (6)	22 (18)	24 (14)	3.453	0.178
Proportion of 6–9 working years (%)	32 (20.5)	31 (18)	31 (26.5)	0.421	0.810
Proportion of more than 10 working years (%)	27 (26)	30 (23)	25 (16.5)	3.668	0.160
Overtime (n,%)					
Absolutely not	8 (12.3)	21 (17.6)	4 (10.8)	3.810	0.432
Very few	39 (60.0)	57 (47.9)	18 (48.6)		
More	18 (27.7)	41 (34.5)	15 (40.5)		
Nursing staff utilization (n,%)					
Insufficient	9 (13.8)	6 (5.0)	0 (0)	10.356	0.035
Sufficient	45 (69.2)	94 (19.0)	27 (73.0)		
Excessive	11 (16.9)	19 (16.0)	10 (27.0)		
Overworked (n,%)					
Absolutely not	35 (53.8)	50 (42.0)	10 (27.0)	8.559	0.051
Very few	28 (43.1)	66 (55.5)	27 (73.0)		
More	2 (3.1)	3 (2.5)	0 (0.0)		
Psychological problems (n,%)					
Absolutely not	50 (76.9)	85 (71.4)	23 (62.2)	2.251	0.287
Very few	15 (23.1)	34 (28.6)	14 (37.8)		
Teamwork (n,%)					
Very good	59 (90.8)	100 (84.0)	33 (89.2)	1.691	0.430
Better	6 (9.2)	19 (16.0)	4 (10.8)		
Satisfaction (n,%)					
Satisfaction	58 (89.2)	103 (86.6)	32 (86.5)	1.853	0.810
General	6 (9.2)	15 (12.6)	4 (10.8)		
Dissatisfaction	1 (1.5)	1 (0.8)	1 (2.7)		

### Correlation analysis between nursing workforce allocation and effect evaluation

We found that the overtime work of nurses was proportional to the average work time per shift (γ_s_ = 0.146, *p* = 0.028) and inversely proportional to the weekly rest time (γ_s_ = −0.195, *p* = 0.003). The utilization of nursing human resources was significantly positively correlated with the proportion of senior nurses (γ_s_ = 0.136, *p* = 0.039). Physical fatigue of nurses was positively correlated with the ratio of nurses with bachelor-level and above degrees (γ_s_ = 0.133, *p* = 0.044), but was significantly negatively correlated with the weekly rest time (γ_s_ = − 0.155, *p* = 0.019). The ratio of nurses with junior college degrees was significantly positively correlated with teamwork (γ_s_ = 0.142, *p* = 0.032), but significantly negatively correlated with the satisfaction of head nurses (γ_s_ = 0.137, *p* = 0.040). The proportion of nurses who have been working for more than 10 years were significantly negatively correlated with both teamwork and psychological problems (γ_s_ = − 0.135, *p* = 0.041; γ_s_ = − 0.203, *p* = 0.002). Additional details regarding this analysis are presented in Tables 3 and 4.

**Table 3:** Assignment of independent variables

Variable	Assignment
Overtime work	not at all = 1; rarely = 2; more = 3
Human resources utilization	insufficient = 1; sufficient = 2; excessive = 3
Have to change jobs or vacations because of physical fatigue	not at all = 1; rarely = 2; more = 3
Have to change jobs or leave because of psychological problems	not at all = 1; rarely = 2; more = 3
Teamwork	very good = 1; better = 2; moderate = 3; bad = 4
Head nurse satisfaction	satisfaction = 1; general = 2; dissatisfaction = 3

**Table 4:** Relationship between nursing workforce allocation and effect evaluation

	Overtime work	Human resources utilization	Physical fatigue	Psychological problems	Teamwork	Satisfaction
Average bed-to-nurse ratio	−0.107	0.052	0.087	0.039	−0.006	− 0.028
Average shift hours (h)	**0.146**^a^	0.055	−0.112	− 0.046	− 0.009	0.035
Average weekly rest time per person (d)	**−0.195**^a^	−0.004	**− 0.155**^a^	− 0.056	− 0.027	0.037
Nurse ratio (%)	0.018	−0.104	− 0.003	− 0.002	0.078	− 0.034
Senior nurse ratio (%)	− 0.071	**0.136**^a^	0.005	0.015	−0.021	−0.071
Supervisor nurse or above ratio (%)	0.115	−0.008	− 0.027	0.041	0.046	−0.013
Junior college degree ratio (%)	0.049	−0.041	−0.068	− 0.052	**0.142**^a^	**− 0.137**^a^
Undergraduate degree or above ratio (%)	−0.021	0.013	**0.133**^a^	−0.031	−0.106	− 0.006
Proportion of 0–5 working years (%)	− 0.052	0.097	− 0.056	0.013	**0.145**^a^	−0.018
Proportion of 6–9 working years (%)	−0.009	0.000	0.000	0.008	0.035	−0.084
Proportion of more than 10 working years (%)	−0.059	−0.107	0.009	**−0.203**^a^	**− 0.135**^a^	−0.018

^a^Statistically significant difference

### Multiple analysis of the effect of nursing workforce allocation

First, we performed a multiple stepwise regression analysis to identify potential predictors of first-line nurses’ satisfaction, yielding the following model equation: Satisfaction = 4.703 - (0.051 × nurse work hours per shift) (see Table 5 for details). This model demonstrated that only the nurse work hours per shift was predictive of nurses’ satisfaction, accounting for 5.2% of the variance in these values. Next, the average length of stay (LOS) of patients was considered as another evaluation index, and we found that the proportion of nurses and the work hours of nurses per shift were both independent influencing factors, and the regression equation was formulated as follows: LOS = 19.581 - (6.72 × nurse ratio) - (0.632 × nurse work hours per shift) (Table 6).

**Table 5:** Multiple stepwise regression analysis of factors influencing satisfaction

Equation	B	S.E	Standardized B	t	P	R^2^
Constant	4.703	0.103		45.550	<0.000	0.052
Nurse work hours per shift	−0.051	0.015	−0.228	−3.515	0.001

**Table 6:** Multiple stepwise regression analysis of factors influencing LOS

Equation	B	S.E	Standardized B	t	P	R^2^
Constant	19.581	2.170		9.024	0.000	0.046
Nurse ratio	−6.72	2.797	−0.158	−2.402	0.017	
Nurse work hours per shift	−0.632	0.280	−0.149	−2.257	0.025	

## Discussion

### Number of nurses in isolation wards

In this study, we found that the bed-to-nurse ratios of the suspected case and confirmed case wards were approximately 1:0.56 and 1:0.45, respectively, and that for the confirmed severe case ward (ICU) was approximately 1:1.88. These results were consistent with those of Cao et al. [11], i.e., the bed-to-nurse ratio of the suspected case and confirmed case wards were both 1: 0.45, while the bed-to-nurse ratio was lower than the reported ratio of 1: ≤ 2.5 in the ICU. Wang et al. [12] refined the allocation of nursing workforce per shift, pointing out that the nurse-to-patient ratio per shift was 1:5 for the suspected cases ward, 1: 3 for the confirmed cases ward, and 1:1 for the ICU. Besides, it was identified that the nurse-to-patient ratio should be 1:1 per shift for patients who have prone position ventilation, invasive mechanical ventilation, or hemodynamic instability. This is consistent worldwide [8]. Therefore, if we encounter a pandemic similar to COVID-19 again, it is suggested that designated hospitals refer to this practical experience for the preliminary allocation of the nursing workforce, and thereafter combine the characteristics of each hospital with the number of patients for a dynamic allocation of the nursing workforce. In summary, the allocation of nursing human resources is the basis for the prevention and control of public health emergencies such as the COVID-19 pandemic. In the future, real-time nurse-to-patient ratios of clinical decision-making systems can be used to appropriately allocate the workforce. Also, based on the preset nurse-to-patient ratio, combined with the number of real-time patients, the reasonable number of nurses and the number of surplus nurses in each ward can be calculated, which can help in the dynamic allocation of the nursing workforce [13].

### Nurse structural configuration in isolation wards

This study found that the structure of nurses in different isolation wards was consistent in the distribution of professional titles and working years, i.e., the professional titles of nurses were dominated by the title of ‘senior nurse’ which accounted for an average of 43 to 50% of all nurse titles. The proportion of nurses working for 0–2 years was less than 10%, while the average distribution was in the medium or many years of service, with 3–5 years of service accounting for about 30%, and ≥ 10 years of service accounting for 5–9%, respectively. The differences were mainly in the educational background of nursing staff. The proportion of nurses with junior college degrees in different wards was different (Z = 8.693, *P* = 0.013). The proportion of nurses with junior college degrees in the suspected case and confirmed case wards were similar (40% vs 38%, *P* = 0.256), while in the ICU ward it was only 20%, and the proportion of nurses with bachelor’s degrees was as high as 73%. This indicates that nurses must have strong professional nursing and isolation protection skills, especially nurses in the ICU, since they are in close contact with patients and provide care for patients with serious conditions or high failure-to-rescue rates. Thus, nursing care during the COVID-19 pandemic is beyond the standard of regular nursing care and has higher requirements for professional literacy [14]. At present, there is a lack of research on nurse structural configurations in various forefronts of the COVID-19 pandemic. The results of this study can provide a reference for hospitals globally on the response to the COVID-19 pandemic and to optimize the mix of skills among nurses.

### Nurse rostering in isolation wards

A good nurse rostering should not only ensure the quality of clinical nursing but also meet the resting needs of nurses, to ensure the safety and continuity of nursing care and maximize the efficiency of nursing workforce allocation and nursing service quality. The results of this study showed that nurses in the suspected case and confirmed case wards worked an average of 6 h per shift, which was higher than that of 109 COVID-19 first-line nurses who worked 5 h per shift, while their desired work hours per shift was 4 h [10]. The average duration of shift for nurses in the ICU ward of confirmed cases in this study was 5 h per shift, which was higher than the 4 h recommended by the National Health Commission of the People’s Republic of China [15]. These numbers illustrate that on the one hand, there is a lack of nurses, while on the other hand, it may be due to the shortage of protective equipment, such as masks and isolation gowns. Hospitals have to extend nurses’ work hours per shift to reduce the use of new protective equipment. However, excessively long work hours will not only lead to increased fatigue and psychological pressures for nurses but may also affect the quality of clinical nursing care. In addition, the results of regression analyses in this study showed that the work hours per shift was the main factor influencing nurses’ satisfaction and the average hospitalization days of patients. Thus, it can be seen that nurse rostering is one of the important indicators of scientific and reasonable allocation of the nursing workforce. Some studies recommend that direct nursing care should be 4 h per shift, while indirect nursing care could be 6 to 8 h per shift [12, 16]. Other hospitals have suggested that the general isolation ward could adopt an 8-h shift and a 4-h shift, inside and outside the isolation area, respectively. Each shift could have a resting time of 2 or 3 days per week. Besides, the ICU could adopt the “4 × 6-1” model of nurse rostering, i.e., each shift would be 4 h a day, for 6 days in a row, with 1 day off [11]. Therefore, nurse managers may attempt to change the general nursing work mode, according to the type and content of the assignment, adjust the work hours of each shift, and implement flexible duty shifts.

### Evaluation of nursing workforce allocation in isolation wards

We found that the overall nursing workforce allocation in the isolation wards of COVID-19 designated hospitals in China is better than stipulated above. For example, the satisfaction of nurses is high, the utilization rate of nursing human resources is sufficient, and the team cooperation is good. However, the problem of long work hours for nurses does exist. In particular, 27.7% of hospitals had nurses working overtime, with nurses working more than 60% of their usual work hours. Correlation analysis identified that the overtime work of nurses was proportional to the average work time per shift (γ_s_ = 0.146) and inversely proportional to the weekly rest time (γ_s_ = − 0.195), which indicates that for longer work hours of nurses, the occurrence of overtime work is more serious. The reason could be heavy workloads or the lack of nursing staff. Therefore, to allocate the nursing workforce effectively, it is necessary to accurately consider the needs of patients, and the number of nurses, and their physical and mental needs, to ensure that nursing care can be conducted continuously and effectively.

We also found that the overall rate of nursing human resource utilization was proportional to the proportion of senior nurses in the ward (r_s_ = 0.136), implying that the greater the proportion of senior nurses in the ward, the higher the utilization rate of nursing human resources, which further illustrates that senior nurses play an important role during the COVID-19 pandemic. In addition, the ratio of nurses with junior college degrees was significantly positively correlated with teamwork (r_s_ = 0.142), which indicates that nurses with junior college degrees were weak in teamwork and communication skills. The ratio of nurses who have worked for more than 10 years was inversely proportional to teamwork and nurses’ psychological problems (r_s_ = 0.135, r_s_ = 0.203), which indicated that nurses with more work experience had stronger abilities for team cooperation, communication skills, and coping skills under pressure. But research conducted by Wang et al. [17] pointed out that the proportion of nurses with work experience of ≤5 years was a protective factor for preventing adverse events. Therefore, the proportion of nurses at all levels should be taken into account in the allocation of the nursing workforce in response to public health emergencies in the future.

## Research limitations

This study has the following limitations: 1) being a retrospective survey, the results of this study may have memory bias; 2) the research is not adequately detailed, such as for the allocation of nursing workforce per shift and the nurse rostering model, etc. This study adopted a self-reported questionnaire of nursing human resource allocation of the isolation wards in COVID-19 designated hospitals during the pandemic, and due to the lack of practical experience and theoretical knowledge, the researchers were not proficient in the design of appropriate investigative tools. Hence, it was difficult to conduct an in-depth exploration of the research results. In the future, it is necessary to improve the quality of investigative tools and conduct an in-depth analysis of nursing human resource management during the COVID-19 pandemic.

## Conclusions

The outbreak of COVID-19 has led to a global economic recession and has seriously threatened population health, social stability, and national public health security. The COVID-19 pandemic may continue in the future, hence the rational allocation of the nursing workforce is the guarantee for sustainable disease prevention and control. Hospitals in China had implemented a rapid response to the COVID-19 pandemic and achieved good results in nursing workforce allocation, but some shortcomings do exist. For instance, the nursing workforce of the first-line isolation ward varies greatly in different hospitals, the allocation of nursing workforce allocation is insufficient, the overall utilization rate of the nursing workforce is at a high level, and the average shift duration is higher than expected. Therefore, it is imperative to establish a scientific and efficient practical scheme for nursing workforce allocation in the future and improve the ability of hospitals to deal with public health emergencies.

## Data Availability

The datasets generated and analysed during the current study are not publicly available because they contain private information about hospitals and patients, but they are available from the corresponding author on reasonable request.

## Abbreviations

COVID-19: Corona Virus Disease 2019
WHO: World Health Organization
ICU: Intensive Care Unit
LOS: Length of stay
